# The role of chemokines in severe malaria: more than meets the eye

**DOI:** 10.1017/S0031182013001984

**Published:** 2013-12-13

**Authors:** LISA J. IOANNIDIS, CATHERINE Q. NIE, DIANA S. HANSEN

**Affiliations:** 1 The Walter and Eliza Hall Institute of Medical Research, 1G Royal Parade, Parkville, Victoria 3052, Australia; 2 Burnet Institute, 85 Commercial Road, Melbourne, Victoria 3004, Australia

**Keywords:** chemokines, severe malaria, pathogenesis

## Abstract

*Plasmodium falciparum* malaria is responsible for over 250 million clinical cases every year worldwide. Severe malaria cases might present with a range of disease syndromes including acute respiratory distress, metabolic acidosis, hypoglycaemia, renal failure, anaemia, pulmonary oedema, cerebral malaria (CM) and placental malaria (PM) in pregnant women. Two main determinants of severe malaria have been identified: sequestration of parasitized red blood cells and strong pro-inflammatory responses. Increasing evidence from human studies and malaria infection animal models revealed the presence of host leucocytes at the site of parasite sequestration in brain blood vessels as well as placental tissue in complicated malaria cases. These observations suggested that apart from secreting cytokines, leucocytes might also contribute to disease by migrating to the site of parasite sequestration thereby exacerbating organ-specific inflammation. This evidence attracted substantial interest in identifying trafficking pathways by which inflammatory leucocytes are recruited to target organs during severe malaria syndromes. Chemo-attractant cytokines or chemokines are the key regulators of leucocyte trafficking and their potential contribution to disease has recently received considerable attention. This review summarizes the main findings to date, investigating the role of chemokines in severe malaria and the implication of these responses for the induction of pathogenesis and immunity to infection.

## INTRODUCTION

Malaria is one the most serious infectious diseases of humans with over 250 million clinical cases every year worldwide. The infection is transmitted to humans by *Anopheles* mosquitoes that are infected with parasites of the genus *Plasmodium*. Most cases of severe disease are caused by *Plasmodium falciparum*, which is endemic in sub-Saharan Africa and throughout the tropics. The blood stage of the parasite is largely responsible for malaria-associated pathology (Miller *et al.* 2002). The fatalities are associated with a range of various disease syndromes including acute respiratory distress, metabolic acidosis, hypoglycaemia, renal failure, anaemia, pulmonary oedema and cerebral malaria (CM) (White and Ho, 1992). This disease syndrome is the most severe complication resulting from *P. falciparum* infection and accounts for nearly 1 million deaths every year (Murray *et al.* 2012). Children under the age of five are particularly susceptible to this condition, which is characterized by convulsions, seizures and coma. Similar to young children, pregnant women (particularly primigravid women) are at an increased risk of infection and might develop placental malaria (PM). This serious complication has been found to be associated with adverse pregnancy outcomes including premature labour, intrauterine growth retardation and low birth weight delivery, and is responsible for more than 75 000 infant deaths each year (McGregor, 1984; Steketee *et al.* 2001; Desai *et al.* 2007).

Mature forms of blood-stage malaria express parasitic proteins on the surface of the infected erythrocyte (such as *P. falciparum* Erythrocyte Membrane Protein 1), which allows them to bind to endothelial cells, sequester in vascular beds and avoid clearance in the spleen. Although the precise mechanisms leading to severe malaria syndromes are not completely understood, it is accepted that sequestration of parasitized red blood cells (pRBC) is a major determinant of disease development. Parasite sequestration is thought to induce obstructions in blood flow resulting in hypoxia and haemorrhages (Miller *et al.* 2002) that appear to be associated with the development of organ-specific syndromes such CM and PM. A large body of literature indicates that in addition to parasite sequestration, inflammatory responses mediated by cytokines such as TNF (Molyneux *et al.* 1993), IFN-*γ* and IL-1*β* (Pongponratn *et al.* 2003) are associated with disease severity in human malaria.

Much useful evidence on the inflammatory mechanisms contributing to the induction of severe malaria has been provided by the *Plasmodium berghei* ANKA model. This rodent infection has many features in common with human disease and is thus the best available model for certain aspects of clinical malaria (Schofield and Grau, 2005; Hansen, 2012). Like in humans, *P. berghei*-ANKA pRBC have been found to accumulate in brains of susceptible mice during infection. Moreover, recent evidence revealed that *P. berghei*-ANKA pRBC adhere to brain microvascular endothelial cells in a VCAM-1-dependent fashion (El-Assaad *et al.* 2013). A large body of work demonstrated that host immune responses elicited during *P. berghei* ANKA infection result in detrimental inflammation and contribute to cerebral disease induction. Host responses mediated by inflammatory cytokines such as TNF (Grau *et al.* 1987), LT-*α* (Engwerda *et al.* 2002), IFN-*γ* (Grau *et al.* 1989) and effector cells such as CD4^+^ (Grau *et al.* 1986; Yanez *et al.* 1996; Villegas-Mendez *et al.* 2012) and CD8^+^ T cells (Belnoue *et al.* 2002; Nitcheu *et al.* 2003), NKT cells (Hansen *et al.* 2003) and NK cells (Hansen *et al.* 2007; Ryg-Cornejo *et al.* 2013) have been shown to contribute to severe malaria in this model.

In addition to pRBC, human post-mortem studies revealed the presence of leucocytes and platelets within the brain microvasculature in a substantial proportion of CM cases (Porta *et al.* 1993; Patnaik *et al.* 1994; Grau *et al.* 2003; Hunt and Grau, 2003; Taylor *et al.* 2004). Interestingly, sequestered monocytes and macrophages were found to be more abundant in CM paediatric patients than in severe malarial anaemia (SMA) patients (Coltel *et al.* 2004). Similarly, although the sequestration of pRBC in the placenta appears to be responsible for initiation of pathology, histological evidence revealed the infiltration of various leucocyte populations including monocytes, macrophages, T cells and granulocytes (Ordi *et al.* 2001). An important intervillositis occurs in PM cases with evident sequestration of both pRBC and leucocytes preventing efficient blood flow across the placenta. Moreover, mononuclear intervillous inflammatory infiltration has been found to be associated with low birth weight and preterm delivery (Ordi *et al.* 1998, 2001).

In experimental mouse models, several leucocyte populations including macrophages, neutrophils, T cells, NK cells and platelets have been found in brain blood vessels of CM-affected mice during infection (Grau *et al.* 1987, 1993; Ma *et al.* 1996; Belnoue *et al.* 2002; Nitcheu *et al.* 2003; Hansen *et al.* 2007; Lundie *et al.* 2008). From these populations, CD8^+^ T cells comprise a high proportion of the brain-sequestered leucocyte pool of *P. berghei*-ANKA-infected mice and appear to mediate CM via a perforin-dependent mechanism (Belnoue *et al.* 2002; Nitcheu *et al.* 2003). Moreover, infection of *β*_2_-microglobulin^−/−^ mice as well as antibody depletion studies demonstrated that CD8^+^ T cells contribute to the induction of experimental cerebral malaria (ECM) (Yanez *et al.* 1996).

The increasing body of evidence illustrating the presence of sequestered host cells together with infected erythrocytes in organs such as the brain or placenta during infection suggested that in addition to secreting cytokines and mediating systemic responses, leucocytes might also contribute to disease by migrating to the site of parasite sequestration, thereby exacerbating organ-specific inflammation. These observations attracted substantial interest in identifying trafficking pathways by which leucocytes are recruited to target organs during severe malaria syndromes and the potential contribution of chemokines to disease. In this review we have summarized the main findings to date investigating the role of chemokines during severe malaria including experimental infection in murine models as well as human field studies. The implications of those findings for the induction of pathogenesis and immunity to malaria are discussed.

## THE ROLE OF CHEMOKINES IN HOMOEOSTASIS AND INFLAMMATION

Chemoattractant cytokines or chemokines are key regulators of leucocyte trafficking. Chemokine-guided movement allows leucocyte migration to various lymphoid tissues and deployment of immune cells to peripheral sites of pathogen challenge and inflammation. Chemokines are a superfamily of low molecular weight polypeptides of about 8–14 kDa, which bind and signal through seven-transmembrane spanning G-protein coupled receptors (GPCRs). To date, nearly 50 chemokines have been identified (Table 1) as well as numerous chemokine-binding proteins that do not induce cell-signalling events (Murphy, 2002). Different chemokines share a significant degree of sequence homology and are subdivided into CC, CXC, CX_3_C and C subfamilies based on the relative positions of conserved cysteine residues near the N-terminus (Luster, 1998).

**Table 1.** Chemokines and chemokine receptors

Cell type	Receptor	Chemokine^a^
Eosinophil Monocyte Activated T cell Dendritic cell	CCR1	**MCP-3 (CCL7), MCP-4 (CCL13), MIP-1*α* (CCL3), RANTES (CCL5), *HCC-1* (*CCL14*)**
Basophil Monocyte Activated T cell Dendritic cell NK cell	CCR2	**MCP-1 (CCL2), MCP-2 (CCL8), MCP-3 (CCL7), MCP-4 (CCL13)**, *MCP-5* (*CCL12*)
Eosinophil Basophil Dendritic cell Activated T cell Resting T cell	CCR3	**Eotaxin-1 (CCL11), Eotaxin-2 (CCL24), MCP-3 (CCL7), MCP-4 (CCL13), RANTES (CCL5), *PARC* (*CCL18*)**
Activated T cell Dendritic cell	CCR4	**TARC (CCL17), MDC (CCL22)**
Monocyte Activated T cell Dendritic cell NK cell	CCR5	**MIP-1*α* (CCL3), MIP-1*β* (CCL4), RANTES (CCL5), *HCC-1* (*CCL14*)**
Dendritic cell	CCR6	*MIP-3α* (*CCL20*)
Activated T cell Resting T cell	CCR7	***MIP-3β* (*CCL19*)**, *SLC* (*CCL21*)
Monocyte	CCR8	**I-309 (CCL1)**
Activated T cell Resting T cell Monocyte Dendritic cell	CCR9	***TECK* (*CCL25*)**
Monocyte activated T cell NK cell	CX_3_CR1	**Fractalkine (CX3CL1)**
Neutrophil	CXCR1	**IL-8 (CXCL8), GCP-2 (CXCL6)**
Neutrophil	CXCR2	**IL-8 (CXCL8), GCP-2 (CXCL6), GRO-*α* (CXCL1), GRO-*β* (CXCL2), GRO-*γ* (CXCL3), ENA-78 (CXCL5), NAP-2 (CXCL7)**
Activated T cell NK cell	CXCR3	**IP-10 (CXCL10), MIG (CXCL9), I-TAC (CXCL11)**
Monocyte Resting T cell Dendritic cell	CXCR4	***SDF-1* (*CXCL12*)**
NK cell Activated T cell Dendritic cell	XCR1	*Lymphotactin* (*XCL1*), *SCM-1β* (*XCL2*)

a Chemokines are identified with both common names and systematic names in parenthesis. Bold font identifies inflammatory/inducible chemokines. Homoeostatic chemokines are shown in italic font. Bold and italic font denotes chemokines belonging to both classifications.

Chemokines and chemokine receptors are essential in two distinct processes of leucocyte migration. The first is the directional migration and positioning of leucocytes within lymphoid organs as well as in peripheral tissues. In this context, cells encounter chemoattractant signals in a complex spatial and temporal pattern, in which cells follow a gradient of increasing concentration towards the source of the chemokine (reviewed in Rot and von Andrian (2004). The second trafficking process mediated by chemokines involves the arrest of migrating leucocytes on the vascular endothelium followed by extravasation from the blood vessel into lymphoid and inflamed tissues. In this process, chemokines are immobilized by sulphated sugars of the glycosaminoglycan (GAG) family on the luminal surface of vascular endothelial cells, which is essential for optimal adhesion of leucocytes to the endothelium (Rot, 1992; Tanaka *et al.* 1993).

Functionally, chemokines can be divided into two broad categories: constitutively expressed chemokines that maintain homoeostatic functions, and inducible chemokines that are usually upregulated in response to infection and/or inflammation (Sallusto *et al.* 2000; Moser *et al.* 2004). This distinction can be somewhat arbitrary as some chemokines fall into both categories depending on the biological context (Table 1). Inflammatory chemokines are produced by several cell types including stromal, endothelial and epithelial cells as well as leucocytes. Constitutive chemokines are produced in the thymus and lymphoid tissues and regulate homoeostatic functions of the immune system such as maintaining steady-state leucocyte homing and cell compartmentalization within lymphoid organs (Yoshie *et al.* 1997). Leucocytes have the capacity to switch the expression of receptors from constitutive to inflammatory chemokines, which allows for different migratory patterns. For example, upon antigen-specific activation and differentiation into effector function, T cells upregulate a repertoire of chemokine receptors and adhesion molecules, which facilitates their exit from secondary lymphoid organs and migration to peripheral inflamed tissues (Xie *et al.* 1999; Weninger *et al.* 2001).

Different effector cell subsets have been shown to express distinct chemokine receptor patterns, allowing them specific migration patterns to exert their function. Polarization of CD4^+^ T cells to T_H_1 and T_H_2 subsets is associated with the upregulation of a distinct set of chemokines and their chemokine receptors, which is influenced by the cytokine milieu during priming (Sallusto *et al.* 1998; Bromley *et al.* 2008). Typically, T_H_1 cells are predominantly characterized by the expression of CXCR3, CCR5 and CXCR6, whereas T_H_2 cells express CCR3, CCR4 and CCR8. However, the association of chemokine receptors with T-helper phenotypes *in vivo* shows a much more complex profile (Annunziato *et al.* 1999; Kim *et al.* 2001), suggesting that CD4^+^ T cell subsets cannot always be accurately identified solely on the basis of their chemokine receptor expression. Moreover, increasing evidence suggests that the chemokine receptor repertoire expressed on activated T cells shows a degree of dynamic plasticity, whereby several factors including strength of antigenic signals, tissue-specific imprinting by priming dendritic cells or cytokine milieu during priming can influence the trafficking phenotype of T cells (reviewed in Mora and von Andrian (2006).

## ASSOCIATION OF CHEMOKINES WITH HUMAN SEVERE MALARIA SYNDROMES

Several inflammatory chemokines have been found to be associated with severe malaria syndromes in case-control studies (Table 2). Increased levels of CXCL8 and CXCL9 as well as reduced levels of CCL5 (also known as RANTES) have been observed in severe malaria patients (Burgmann *et al.* 1995; Ochiel *et al.* 2005; Ayimba *et al.* 2011; Lopera-Mesa *et al.* 2012). Moreover, CXCL8 and CXCL9 levels have been shown to correlate with parasite density (Burgmann *et al.* 1995; Ayimba *et al.* 2011) suggesting that increased parasitic loads might induce these responses. In support of that concept, *in vitro* studies demonstrated that *P. falciparum*-infected erythrocytes are able to induce CCL2, CCL20, CXCL1, CXCL2, CXCL6 and CXCL8 production by human endothelial cells (Viebig *et al.* 2005; Chakravorty *et al.* 2007; Tripathi *et al.* 2009).

**Table 2.** Association between chemokines and the outcome of human malaria infections

Chemokine	Production during infection	Association	Reference
CXCL8	Increased in the serum and plasma of patients with severe malaria	Serum CXCL8 levels were shown to correlate with parasite density	(Burgmann *et al.* 1995; Lopera-Messa *et al.* 2012)
	Increased in the CSF of children with CM	CSF CXCL8 levels were independently predictive of CM mortality	(Armah *et al.* 2007; John *et al.* 2008*a*, *b*)
	Increased in the placentae of women with PM	Placental CXCL8 levels were associated with placental monocyte infiltration and adverse pregnancy outcomes	(Abrams *et al.* 2003)
CXCL9	Increased in the plasma of patients with severe malaria	Serum CXCL9 levels were shown to correlate with parasite density	(Ayimba *et al.* 2011)
	Increased in the placentae and peripheral blood of women with PM	Placental CXCL9 levels were negatively associated with low birth weight	(Muehlenbachs *et al*. 2007; Dong *et al.* 2012)
CCL5 (RANTES)	Reduced in the serum of patients with severe malaria	Reduced RANTES levels were associated with suppression of erythropoiesis and thrombocytopenia	(Ochiel *et al.* 2005; Were *et al.* 2006)
	Increased mRNA expression in brain samples from CM patients	Not determined	(Sarfo *et al.* 2004)
CCL4	Increased in the plasma of children with CM	Not determined	(Jain *et al.* 2008)
	Increased in the CSF of children with CM	CSF CCL4 levels were independently predictive of CM mortality	(Armah *et al.* 2007)
	Increased in the placentae of women with PM	Placental plasma CCL4 levels were associated with low birth weight	(Abrams *et al.* 2003; Muehlenbachs *et al*. 2007)
CXCL10	Increased in the serum and plasma of children with CM	Circulating IP-10 levels were independently associated with CM mortality	(Armah *et al.* 2007; Jain *et al.* 2008; Wilson *et al.* 2011)
	Increased in the CSF of children with CM	Not determined	(Armah *et al.* 2007)
	Increased in the placentae and peripheral blood of women with PM	Not determined	(Muehlenbachs *et al*. 2007; Boström *et al.* 2012)
CCL2	Increased in the placentae and peripheral blood of women with PM	Placental CCL2 levels were associated with placental monocyte infiltration	(Abrams *et al.* 2003; Dong *et al.* 2012)
CCL3	Increased in the placentae and peripheral blood of women with PM	Placental and peripheral blood CCL3 levels were associated with placental monocyte infiltration	(Abrams *et al.* 2003)
CXCL13	Increased in the placentae and peripheral blood of women with PM	Placental CXCL13 levels negatively correlated with low birth weight	(Muehlenbachs *et al*. 2007; Dong *et al.* 2012)
CXCL16	Increased in the placentae of women with PM	Not determined	(Muehlenbachs *et al*. 2007)

Severe malarial anaemia is one of the most common clinical manifestations of disease in children (Marsh *et al.* 1995). Both erythropoietic suppression (Kurtzhals *et al.* 1997) and RBC destruction (Looareesuwan *et al.* 1991) are thought to contribute to the development of SMA. Reduced circulating levels of CCL5 have been found to be associated with SMA in children (Ochiel *et al.* 2005; Were *et al.* 2006). Moreover, reduced CCL5 production was associated with the suppression of erythropoiesis and malaria-induced thrombocytopaenia (Were *et al.* 2006), suggesting that CCL5 may be involved in the regulation of the erythorpoietic response during malaria infection. It is thought that monocytic-acquisition of *P. falciparum* hemozoin (*pf*Hz) may in part contribute to CCL5 suppression during SMA, as children with high levels of intramonocytic *pf*Hz have been shown to have lower circulating CCL5 levels than children with only low levels of intramonocytic *pf*Hz (Were *et al.* 2009). This is further supported by the observation that CCL5 production in IFN-*γ*-stimulated PBMCs from malaria-infected children decreased with increasing levels of intramonocytic *pf*HZ (Were *et al.* 2009).

Although reduced circulating levels of CCL5 have been shown to be associated with CM (John *et al.* 2006), increased transcription of this chemokine has been observed in brain samples from CM patients (Sarfo *et al.* 2004). Circulating levels of CCL4 (also known as MIP-1*β*), CXCL10 (also known as IP-10), CXCL4 and CXCL8 have been found to be significantly elevated in CM cases compared with mild malaria cases or healthy control individuals (John *et al.* 2006, 2008*b*; Armah *et al.* 2007; Jain *et al.* 2008; Wilson *et al.* 2011). Amongst these chemokines, CXCL10 appears to be the most accurate predictor of CM mortality (Jain *et al.* 2008; Wilson *et al.* 2011). Moreover, cerebrospinal fluid levels of CXCL10, CXCL8 and CCL4 have been found to be significantly higher in children with CM compared with children with SMA and non-malaria-infected individuals (Armah *et al.* 2007; John *et al.* 2008*a*).

## CHEMOKINES AND HUMAN PLACENTAL MALARIA

Most of the available information to date on the role of chemokines in the pathogenesis of severe malaria in humans has been obtained from PM studies. During this serious pregnancy complication, parasite sequestration appears to induce secretion of pro-inflammatory cytokines and chemokines as well as the recruitment of macrophages and monocytes to the intervillous space (Rogerson *et al.* 2003*a*, *b*). Consistent with these observations, increased levels of CCL2 (also known as MCP-1), CCL3 (also known as MIP-1*α*), CCL4, CXCL8, CXCL9, CXCL13 and CXCL16 have been observed in the placentae of women with PM (Abrams *et al.* 2003; Chaisavaneeyakorn *et al.* 2003; Muehlenbachs *et al.* 2007; Dong *et al.* 2012). Reduced levels of CCL5 and increased levels of CXCL10, CXCL9 and CCL2 have also been observed in the peripheral blood of women with PM (Bostrom *et al.* 2012; Dong *et al.* 2012). However, only placental CCL4, CXCL8, CXCL9 and CXCL13 have been associated with adverse pregnancy outcomes such as low birth weight deliveries (Abrams *et al.* 2003; Muehlenbachs *et al.* 2007; Dong *et al.* 2012).

The *β*-chemokines CCL2, CCL3 and CCL4 are chemo-attractants for monocytes (Rollins, 1997), while CXCL8 is thought to promote monocyte adhesion (Gerszten *et al.* 1999). Thus, production of these chemokines in the placenta during infection may promote monocyte infiltration of the intervillous space. Consistent with this idea, it has been reported that placental CCL2, CCL3 and CXCL8 levels are associated with placental monocyte infiltration (Abrams *et al.* 2003). The intravillous infiltrate primarily consists of monocytes and macrophages. Small numbers of B cells, T cells and granulocytes have also been observed in this infiltrate (Ordi *et al.* 2001). Although it is unclear how these cells migrate to the intervillous space during infection, it is possible that recruitment of B and T cells may be mediated by CXCL13 and CXCL9 respectively.

Both maternal and fetal cells appear to contribute to chemokine production in the placenta during PM. Histopathological analysis of malaria-infected placentae has shown that maternal macrophages and fetal stromal cells contribute to CCL3, CCL4 and CXCL13 production in this organ (Abrams *et al.* 2003; Muehlenbachs *et al.* 2007), while CCL2, CCL3, CCL4 and CXCL10 have been found in intervillous blood monocular cells isolated from malaria-infected placentae (Suguitan *et al.* 2003). In addition, *in vitro* differentiated syncytiotrophoblasts have been shown to produce CCL3, CCL4 and CXCL8 in response to stimulation with malaria hemozoin (Lucchi *et al.* 2008, 2011), which is highly abundant in the placenta during PM (Galbraith *et al.* 1980).

## THE ROLE OF CHEMOKINES IN SEVERE DISEASE AND IMMUNITY: LESSONS FROM MOUSE INFECTION MODELS

The initial observations supporting a role for inflammatory chemokines in the aetiology of ECM were provided by gene expression studies. Microarray analysis revealed that the expression of CCR5, CXCR3 and CCR1 and several of their chemokines including CXCL9, CXCL10, CCL2, CCL3 and CCL9 significantly increases in response to infection in CM-affected mice (Sexton *et al.* 2004; Hansen *et al.* 2005). CCL5, CCR1, CCR3 and CCR5 mRNA expression was also detected in brains of Swiss Webster mice and appeared to contribute to the inflammatory response that results in cellular degradation in the cerebellum during *Plasmodium yoelii* (17XL) infection (Sarfo *et al.* 2005). Further chemokine gene expression studies in brains of ECM-susceptible mice confirmed that CXCL10, CXCL9 and CCL5 and to lesser extent CCL2, CCL7, CCL3, CCL4 and CXCL2 are upregulated during infection with *P. berghei* ANKA (Hanum *et al.* 2003; Campanella *et al.* 2008; Miu *et al.* 2008; Van den Steen *et al.* 2008). Interestingly, the expression of CCL2, CCL5, CCL4, CXCL9 and CXCL10 is induced by either IFN-*γ* or TNF, which is consistent with the important role of these pro-inflammatory cytokines in ECM pathogenesis.

To study the mechanism of leucocyte recruitment to the brain during malaria infection several studies analysed the chemokine receptor usage of brain-sequestered leucocytes from infected mice. CD8^+^ T cells isolated from the spleen and brain of malaria-infected mice significantly upregulated CCR5, CCR2, CXCR4 and CXCR3 expression (Nitcheu *et al.* 2003), initially suggesting that trafficking via these chemokine receptors could be involved in the recruitment of inflammatory cells during severe disease. However, further studies indicated that CCR2 deficient mice (Table 3) are fully susceptible to ECM (Belnoue *et al.* 2003*a*). Although there has been conflicting evidence on the role of CCR5 in ECM with CCR5^−/−^ mice reported to be either 80% resistant to *P. berghei* ANKA-mediated CM (Belnoue *et al.* 2003*b*) or to display only a delayed onset of cerebral disease symptoms (Nitcheu *et al.* 2003), mixed-*Plasmodium* species infection experiments support a role for this trafficking pathway in CM. *Plasmodium berghei* ANKA-induced CM is inhibited by the co-infection with the non-virulent *P. yoelii yoelii* 17X clone 1.1 (Table 3). Protection was found to be associated with reduced accumulation of CD8^+^ T cells in the brain vasculature as well as reduced CCL3, CCL4 and CCL5 levels in the brain (Clark and Phillips, 2011).

**Table 3.** Effect of genetic deletion or neutralization of chemokines/chemokine receptors on the outcome of malaria infection in rodent models

Chemokine/chemokine receptor	Parasite	Mouse strain	Effect on disease	Reference
CCR2	*P. berghei*-ANKA	CCR2^−/−^	CCR2^−/−^ mice were fully susceptible to CM	(Belnoue *et al.* 2003*a*)
	*P. chabaudi adami*	CCR2^−/−^	No effect	(Weidnaz *et al.* 2010)
	*P. chabaudi chabaudi* AS	CCR2^−/−^	Parasite clearance was delayed and monocyte recruitment to the spleen was reduced in CCR2^−/−^ mice	(Sponaas *et al.* 2009)
CCR5	*P. berghei*-ANKA	CCR5^−/−^	CCR5^−/−^ mice were shown to be 80% resistant to CM	(Belnoue *et al.* 2003*b*)
	*P. berghei*-ANKA	CCR5^−/−^	CCR5^−/−^ mice only showed delayed onset of cerebral disease	(Nitcheu *et al.* 2003)
CCL3 CCL4 CCL5	*P. berghei* ANKA*/ P. yoelii yoelii* 17X clone 1.1		*P. berghei* ANKA-mediated CM was inhibited by the co-infection *P. yoelii yoelii* 17X clone 1.1. Protection was associated with reduced CCL3, CCL4 and CCL5 levels in the brain	(Clark and Phillips, 2011)
CXCL9	*P. berghei*-ANKA	CXCL9^−/−^	CXCL9^−/−^ mice were partially protected from CM	(Campanella *et al.* 2008)
CXCL10	*P. berghei*-ANKA	CXCL10^−/−^	CXCL10^−/−^ mice had reduced cerebral intravascular inflammation and peripheral parasitaemia, and were protected from CM	(Nie *et al.* 2009)
	*P. berghei*-ANKA	C57BL/6	CXCL10 neutralization during infection reduced cerebral intravascular inflammation and peripheral parasitaemia, and protected mice from CM	(Nie *et al.* 2009)
	*P. berghei*-ANKA	C57BL/6	Pharmacological inhibition of CXCL10 in combination with anti-malarial therapy protected mice from CM	(Wilson *et al.* 2013)
CXCL4	*P. berghei*-ANKA	PAF4^−/−^	PAF4^−/−^ mice were partially protected from CM and showed reduced T cell infiltration into the brain during infection	(Srivastava *et al.* 2008)
CXCR4	*P. chabaudi* CR	C57BL/6	CXCR4 blockade resulted in increased recrudescent parasitaemias	(Garnica *et al.* 2002)

Further phenotypic characterization of leucocytes isolated from brain blood vessels of malaria-infected mice by flow cytometry revealed that 80–90% of NK cells and T cells expressed CXCR3, indicating that the expression of this chemokine receptor is strongly associated with lymphocyte trafficking during CM (Hansen *et al.* 2007). Moreover, *in vitro* chemotaxis assays revealed that whereas T cells from naive mice were unable to migrate in response to the CXCR3-ligand CXCL10, T cells from *P. berghei* ANKA-infected mice showed a 3-fold increase in their CXCL10-mediated chemotaxis (Hansen *et al.* 2007), indicating that during malaria, T cells acquire the capacity to migrate in response to this chemokine. This appears to be a feature specific of CM-susceptible mouse strains, as T cells from malaria-infected CM-resistant BALB/c animals were found to be unable to respond to CXCL10 chemotactic stimulus (Van den Steen *et al.* 2008). Together, this evidence supports a role for CXCR3-mediated trafficking in the migration of pathogenic T cells to the brain during ECM. In agreement, it has been found that 70–80% of CXCR3^−/−^ mice are resistant to *P. berghei*-mediated CM (Campanella *et al.* 2008; Miu *et al.* 2008).

The relative involvement of the 3 CXCR3 chemokines (CXCL9, CXCL10 and CXCL11) in the recruitment of inflammatory leucocytes to the brain of malaria-infected mice has been extensively investigated (Table 3). As CXCL11 is not normally expressed in CM-susceptible C57BL/6 mice (usually used in ECM experiments) a role for this chemokine in this model is not expected. CXCL9^−/−^ mice were found to be partially protected from *P. berghei*-ANKA-mediated CM (Campanella *et al.* 2008). However, whether the increased survival rates to malaria infection in the absence of this chemokine result from reduced leucocyte recruitment to the brain and/or a differential induction of immune response to infection has not been examined.

The role of the CXCR3 chemokine CXCL10 has been studied in more detail. CXCL10^−/−^ mice or mice receiving anti-CXCL10 neutralizing antibodies over the course of *P. berghei*-ANKA infection were found to have reduced cerebral intravascular inflammation and were protected against fatality (Nie *et al.* 2009). Moreover, pharmacological inhibition of CXCL10 together with anti-malarial therapy also protects infected animals from CM-mediated mortality (Wilson *et al.* 2013). Interestingly, CXCL10 blockade during infection resulted in significantly reduced peripheral parasitaemia, suggesting that the absence of this chemokine has a beneficial effect for the development of parasite-specific responses involved in the control of parasite replication (Nie *et al.* 2009). Since *Plasmodium* spp. are blood-borne parasites, the spleen constitutes a key site in the initiation of immune responses and control of parasite replication (Looareesuwan *et al.* 1993; Sayles *et al.* 1993; Yap and Stevenson, 1994; Chotivanich *et al.* 2002). Moreover, it has been proposed that the spleen is the site of initial induction of inflammatory cells that migrate to the site of parasite sequestration in target organs (Renia *et al.* 2006). Consistent with that concept, the increased resistance to infection observed in the absence of CXCL10-mediated cell trafficking was found to be associated with a preferential accumulation and subsequent expansion of parasite-specific CD4^+^ T cells in spleens of infected animals (Nie *et al.* 2009). Overall these findings are consistent with two concepts: (1) trafficking pathways might control the balance between pathogenic organ-specific inflammation and spleen-mediated protective immunity and (2) inflammatory processes that occur during infection are not only detrimental for their involvement in severe disease but can also compromise the induction of anti-parasite immunity by inducing leucocyte migration away from the spleen.

The cellular sources responsible for the secretion of CXCR3 chemokines during ECM have not been extensively investigated, with only a few studies focusing primarily on brain tissue. Gene expression analysis (Miu *et al.* 2008) as well as immunohistochemistry approaches (Campanella *et al.* 2008) revealed that CXCL9 is predominantly expressed by endothelial cells and surrounding microglial cells. There is conflicting evidence on the cellular sources of CXCL10 during infection. Whereas CXCL10 RNA was found to be upregulated in brain endothelial cells and astrocytes clustering around IP-10 positive microvessels (Miu *et al.* 2008), immunohistochemistry studies showed CXCL10 staining mainly in neurons throughout the brain parenchyma and only occasionally on endothelial cells, but not on astrocytes (Campanella *et al.* 2008). Whereas the endothelial and glial localization of CXCL9 and CXCL10 producing cells is consistent with a role for these chemokines in the recruitment of inflammatory leucocytes to the site of parasite sequestration (intravascular infiltration without extravasation into the brain parenchyma), further research is required to determine the effect that CXCL10 upregulation might have in neuronal tissue and the effector signals responsible for this response during infection.

In addition to CXCL9 and CXCL10, the platelet-derived chemokine, platelet factor 4 (PF4 or CXCL4), which binds CXCR3B, a spliced variant of the chemokine receptor CXCR3, was found to be induced in the brain during *P. berghei-*ANKA infection (Srivastava *et al.* 2008). Furthermore, PF4^−/−^ mice showed reduced ECM mortality and T cell infiltration into the brain during infection (Srivastava *et al.* 2008). Thus CXCL4 might contribute to the recruitment of T cells to the brain in ECM pathogenesis. In addition, CXCL4 was also found to enhance monocyte activation in response to *P. berghei*-ANKA infection (Srivastava *et al.* 2010). The precise contribution of this response to disease severity requires further investigation.

Chemokines are not only mediators of cell trafficking during inflammation but are also involved in the recruitment of immune cells to secondary lymphoid organs whereby they enhance the development of protective responses to infection. A few studies have addressed the role that chemokines play in the control of parasite densities using rodent malaria infections. CCR2 is a receptor involved in the recruitment of various cells including activated T cells, NK cells, dendritic cells and monocytes. Whereas genetic deficiency of this chemokine does not appear to affect parasite growth during *P. berghei-*ANKA (Belnoue *et al.* 2003*a*) or *Plasmodium chabaudi adami* infection (Weidanz *et al.* 2010), parasite clearance was found be delayed in CCR2^−/−^ mice infected with *Plasmodium chabaudi chabaudi* AS (Sponaas *et al.* 2009). CCR2 is involved in the recruitment of bone marrow-derived inflammatory monocytes in response to infection (Serbina *et al.* 2008). The reduced control of parasitaemia observed in CCR2^−/−^ mice correlated with significantly lower numbers of inflammatory monocytes in the spleen of these animals, suggesting these cells play a role in controlling blood-stage malaria. Monocytes as well as dendritic cells are also capable of migrating in response to CXCL12, which recognizes the CXCR4 receptor. CXCR4 blockade during *Plasmodium chabaudi* CR infection has also been shown to result in increased recrudescent parasitaemias (Garnica *et al.* 2002), however the precise mechanism by which this trafficking pathway modulates parasite density in this model requires further investigation.

## CONCLUDING REMARKS

Over the past few decades, most of the work on soluble factors associated with the development of severe malaria has mainly focused on the analysis of cytokine responses both in human studies and rodent infection models. The role of chemokines in severe disease induction has only recently started to become appreciated and receive more attention. Cytokines are primarily secreted by immune cells and their main function is modulation of immune responses. Unlike cytokines, chemokines are secreted by a broad range of cellular sources in lymphoid organs and/or inflamed tissue and they appear to control a range of diverse processes including leucocyte trafficking, angiogenesis and tissue remodelling. It is then possible that the cellular sources of chemokines produced in response to malaria differ in inflamed tissue and lymphoid organs and that they control different processes. Further research is needed to fully appreciate the contribution of these chemokine-mediated processes to severe malaria and the precise mechanisms by which inflammatory chemokines exacerbate disease.

**Fig. 1.**
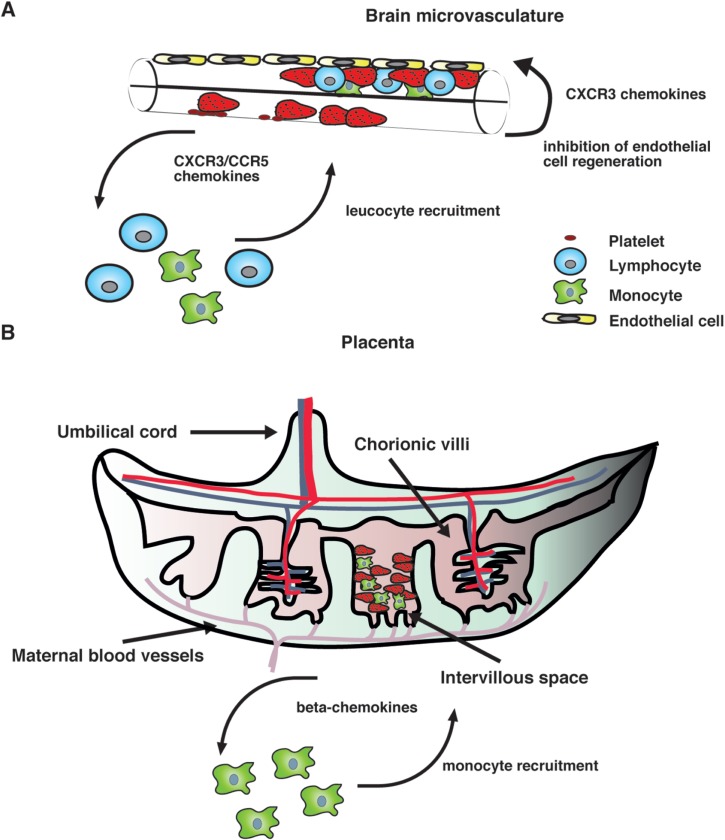
A hypothetical model of action of chemokines in human severe malaria syndromes. (A) After binding to the brain microvasculature, sequestered pRBC induce activation of vascular endothelial cells, which results in the release of inflammatory cytokines as well as CXCR3 and CCR5 binding chemokines. It is possible that local production of these chemokines stimulates the accumulation of CXCR3^+^ and CCR5^+^ leucocytes. In addition, some CXCR3 chemokines such as CXCL10 that have angiostatic activity could inhibit endothelial cell regeneration of the brain microvasculature, thereby compromising the integrity of the blood–brain barrier. (B) In the placenta, both maternal and fetal cells might contribute to the production of *β*-chemokines in response to infection. These mediators stimulate the recruitment of monocytes and macrophages to the intravillous space, which appears to be associated with adverse pregnancy outcomes.

Our current knowledge on human PM supports the idea that the intravillous infiltrate that accompanies parasite sequestration mainly consists of monocytes and macrophages. Consistent with this observation, elevated levels of monocyte-attracting *β*-chemokines such as CCL2 and CCL3 have been found in infected placentae and their production appears to be associated with cellular infiltration (Abrams *et al.* 2003), strongly suggesting that chemokine secretion in the placenta during infection recruits monocytes to the intervillous space (Fig. 1).

On the other hand, mainly CXCR3 and CCR5 binding chemokines have been found to be associated with CM severity in humans (Armah *et al.* 2007). Although the precise mechanism by which these chemokines contribute to human disease remains unclear, it is possible that their local production in the brain during infection might stimulate the accumulation of CXCR3^+^ and CCR5^+^ leucocytes (Fig. 1). Consistent with this hypothesis, increased CXCR3 and CCR5 mRNA levels have been observed in brain samples from children that have succumbed to *P. falciparum*-mediated CM (Sarfo *et al.* 2004). In addition to their chemotactic function, some inflammatory chemokines such as CXCL10 have been shown to exert angiostatic activity (Angiolillo *et al.* 1995; Luster *et al.* 1995; Romagnani *et al.* 2001). It is then possible that locally produced CXCL10 may inhibit endothelial cell regeneration of the brain microvasculature, thereby compromising the integrity of the blood–brain barrier (Fig. 1). In support of this view, the angiogenic factor VEGF has been found to be protective against CM-associated mortality (Armah *et al.* 2007; Jain *et al.* 2008).

Although rodent malaria infection models are not perfect, CXCR3 and CCR5 chemokines (such as CXCL10 and CCL4) that were identified as biomarkers of CM in humans (Armah *et al.* 2007) have also been found to participate in ECM induction in mice (Campanella *et al.* 2008; Nie *et al.* 2009). Thus animal models appear to provide a fast and cost-effective resource to investigate chemokine-mediated mechanisms of disease and for assessment of potential therapeutic interventions (Nie *et al.* 2009; Wilson *et al.* 2013). Moreover, good mouse models of SMA and PM have been developed (Evans *et al.* 2006; Neres *et al.* 2008; Rodrigues-Duarte *et al.* 2012) and are now available to explore the involvement of chemokines in these disease syndromes.

Current views in the field propose that the same inflammatory responses that contribute to severe malaria might be also involved in the control of parasitaemia. This concept has discouraged the use of anti-cytokine treatments (van Hensbroek *et al.* 1996) as adjunctive therapy for complicated malaria cases as it might reduce inflammation but also result in immunosuppression, which could be detrimental for the control of infection. Anti-chemokine therapies (Nie *et al.* 2009) are emerging as potential safe therapeutic alternatives to improve outcomes of severe malaria cases during treatment with anti-malarial drugs, as they alleviate organ-specific inflammation without inducing a generalized immunosuppression of the host.

## FINANCIAL SUPPORT

Supported by the Australian Government National Health and Medical Research Council IRIISS and Project Grant 1031212.

## References

[ref1] Abrams E. T., Brown H., Chensue S. W., Turner G. D. H., Tadesse E., Lema V. M., Molyneux M. E., Rochford R., Meshnick S. R. and Rogerson S. J. (2003). Host response to malaria during pregnancy: placental monocyte recruitment is associated with elevated beta chemokine expression. Journal of Immunology 170, 2759–2764 10.4049/jimmunol.170.5.2759 12594307

[ref2] Angiolillo A. L., Sgadari C., Taub D. D., Liao F., Farber J. M., Maheshwari S., Kleinman H. K., Reaman G. H. and Tosato G. (1995). Human interferon-inducible protein-10 is a potent inhibitor of angiogenesis *in vivo*. Journal of Experimental Medicine 182, 155–162. 10.1084/jem.182.1.155 7540647 PMC2192108

[ref3] Annunziato F., Cosmi L., Galli G., Beltrame C., Romagnani P., Manetti R., Romagnani S. and Maggi E. (1999). Assessment of chemokine receptor expression by human Th1 and Th2 cells *in vitro* and *in vivo*. Journal of Leukocyte Biology 65, 691–699 10331500 10.1002/jlb.65.5.691

[ref4] Armah H. B., Wilson N. O., Sarfo B. Y., Powell M. D., Bond V. C., Anderson W., Adjei A. A., Gyasi R. K., Tettey Y., Wiredu E. K., Tongren J. E., Udhayakumar V. and Stiles J. K. (2007). Cerebrospinal fluid and serum biomarkers of cerebral malaria mortality in Ghanaian children. Malaria Journal 6, 147. 10.1186/1475-2875-6-147 PMC2186349 17997848

[ref5] Ayimba E., Hegewald J., Segbena A. Y., Gantin R. G., Lechner C. J., Agosssou A., Banla M. and Soboslay P. T. (2011). Proinflammatory and regulatory cytokines and chemokines in infants with uncomplicated and severe *Plasmodium falciparum* malaria. Clinical and Experimental Immunology 166, 218–226. 10.1111/j.1365-2249.2011.04474.x 21985368 PMC3219897

[ref6] Belnoue E., Kayibanda M., Vigario A. M., Deschemin J. C., van Rooijen N., Viguier M., Snounou G. and Renia L. (2002). On the pathogenic role of brain-sequestered *αβ* CD8^+^ T cells in experimental cerebral malaria. Journal of Immunology 169, 6369–6375 10.4049/jimmunol.169.11.6369 12444144

[ref7] Belnoue E., Costa F. T., Vigario A. M., Voza T., Gonnet F., Landau I., Van Rooijen N., Mack M., Kuziel W. A. and Renia L. (2003 *a*). Chemokine receptor CCR2 is not essential for the development of experimental cerebral malaria. Infection and Immunity 71, 3648–3651 12761155 10.1128/IAI.71.6.3648-3651.2003 PMC155705

[ref8] Belnoue E., Kayibanda M., Deschemin J. C., Viguier M., Mack M., Kuziel W. A. and Renia L. (2003 *b*). CCR5 deficiency decreases susceptibility to experimental cerebral malaria. Blood 101, 4253–4259 12560237 10.1182/blood-2002-05-1493

[ref9] Boström S., Ibitokou S., Oesterholt M., Schmiegelow C., Persson J.-O., Minja D., Lusingu J., Lemnge M., Fievet N., Deloron P., Luty A. J. F. and Troye-Blomberg M. (2012). Biomarkers of *Plasmodium falciparum* infection during pregnancy in women living in Northeastern Tanzania. PLoS ONE 7, e48763. 10.1371/journal.pone.0048763 23155405 PMC3498253

[ref10] Bromley S. K., Mempel T. R. and Luster A. D. (2008). Orchestrating the orchestrators: chemokines in control of T cell traffic. Nature Immunology 9, 970–980. 10.1038/ni.f.213 18711434

[ref11] Burgmann H., Hollenstein U., Wenisch C., Thalhammer F., Looareesuwan S. and Graninger W. (1995). Serum concentrations of MIP-1*α* and IL-8 in patients suffering from acute *Plasmodium falciparum* malaria. Clinical Immunology and Immunopathology 76, 32–36. 10.1006/clin.1995.1084 7606866

[ref12] Campanella G. S., Tager A. M., El Khoury J. K., Thomas S. Y., Abrazinski T. A., Manice L. A., Colvin R. A. and Luster A. D. (2008). Chemokine receptor CXCR3 and its ligands CXCL9 and CXCL10 are required for the development of murine cerebral malaria. Proceedings of the National Academy of Sciences USA 105, 4814–4819 10.1073/pnas.0801544105 PMC2290783 18347328

[ref13] Chaisavaneeyakorn S., Moore J. M., Mirel L., Othoro C., Otieno J., Chaiyaroj S. C., Shi Y. P., Nahlen B. L., Lal A. A. and Udhayakumar V. (2003). Levels of macrophage inflammatory protein 1*α* (MIP-1*α*) and MIP-1*β* in intervillous blood plasma samples from women with placental malaria and human immunodeficiency virus infection. Clinical and Diagnostic Laboratory Immunology 10, 631–636. 10.1128/cdli.10.4.631-636.2003 12853396 PMC164254

[ref14] Chakravorty S. J., Carret C., Nash G. B., Ivens A., Szestak T. and Craig A. G. (2007). Altered phenotype and gene transcription in endothelial cells, induced by *Plasmodium falciparum*-infected red blood cells: pathogenic or protective? International Journal for Parasitology 37, 975–987. 10.1016/j.ijpara.2007.02.006 17383656 PMC1906861

[ref15] Chotivanich K., Udomsangpetch R., McGready R., Proux S., Newton P., Pukrittayakamee S., Looareesuwan S. and White N. J. (2002). Central role of the spleen in malaria parasite clearance. Journal of Infectious Diseases 185, 1538–1541 11992295 10.1086/340213

[ref16] Clark C. J. and Phillips R. S. (2011). Cerebral malaria protection in mice by species-specific *Plasmodium* co-infection is associated with reduced CC chemokine levels in the brain. Parasite Immunology 33, 637–641. 10.1111/j.1365-3024.2011.01329.x 21851365

[ref17] Coltel N., Combes V., Hunt N. H. and Grau G. E. (2004). Cerebral malaria – a neurovascular pathology with many riddles still to be solved. Current Neurovascular Research 1, 91–110. 10.2174/1567202043480116 16185187

[ref18] Desai M., ter Kuile F. O., Nosten F., McGready R., Asamoa K., Brabin B. and Newman R. D. (2007). Epidemiology and burden of malaria in pregnancy. Lancet Infectious Diseases 7, 93–104. 10.1016/s1473-3099(07)70021-x 17251080

[ref19] Dong S., Kurtis J. D., Pond-Tor S., Kabyemela E., Duffy P. E. and Fried M. (2012). CXCL9 response to malaria during pregnancy is associated with low-birth-weight deliveries. Infection and Immunity 80, 3034–3038. 10.1128/iai.00220-12 22689822 PMC3418745

[ref20] El-Assaad F., Wheway J., Mitchell A. J., Lou J., Hunt N. H., Combes V. and Grau G. E. (2013). Cytoadherence of *Plasmodium berghei*-infected red blood cells to murine brain and lung microvascular endothelial cells *in vitro*. Infection and Immunity. 10.1128/iai.00428-13 PMC3811819 23940206

[ref21] Engwerda C. R., Mynott T. L., Sawhney S., De Souza J. B., Bickle Q. D. and Kaye P. M. (2002). Locally up-regulated lymphotoxin *α*, not systemic tumor necrosis factor *α*, is the principle mediator of murine cerebral malaria. Journal of Experimental Medicine 195, 1371–1377 12021316 10.1084/jem.20020128 PMC2193758

[ref22] Evans K. J., Hansen D. S., van Rooijen N., Buckingham L. A. and Schofield L. (2006). Severe malarial anemia of low parasite burden in rodent models results from accelerated clearance of uninfected erythrocytes. Blood 107, 1192–1199 16210332 10.1182/blood-2005-08-3460 PMC1895912

[ref23] Galbraith R. M., Faulk W. P., Galbraith G. M. P., Holbrook T. W. and Bray R. S. (1980). Human materno-foetal relationship in malaria. 1. Identification of pigment and parasites in the placenta. Transactions of the Royal Society of Tropical Medicine and Hygiene 74, 52–60. 10.1016/0035-9203(80)90011-5 6159702

[ref24] Garnica M. R., Souto J. T., Silva J. S. and de Andrade H. F. Jr. (2002). Stromal cell derived factor 1 synthesis by spleen cells in rodent malaria, and the effects of *in vivo* supplementation of SDF-1*α* and CXCR4 receptor blocker. Immunology Letters 83, 47–53 12057854 10.1016/s0165-2478(02)00067-6

[ref25] Gerszten R. E., Garcia-Zepeda E. A., Lim Y. C., Yoshida M., Ding H. A., Gimbrone M. A., Luster A. D., Luscinskas F. W. and Rosenzweig A. (1999). MCP-1 and IL-8 trigger firm adhesion of monocytes to vascular endothelium under flow conditions. Nature 398, 718–723 10227295 10.1038/19546

[ref26] Grau G. E., Piguet P. F., Engers H. D., Louis J. A., Vassalli P. and Lambert P. H. (1986). L3T4^+^ T lymphocytes play a major role in the pathogenesis of murine cerebral malaria. Journal of Immunology 137, 2348–2354 3093572

[ref27] Grau G. E., Fajardo L. F., Piguet P. F., Allet B., Lambert P. H. and Vassalli P. (1987). Tumor necrosis factor (cachectin) as an essential mediator in murine cerebral malaria. Science 237, 1210–1212 3306918 10.1126/science.3306918

[ref28] Grau G. E., Heremans H., Piguet P. F., Pointaire P., Lambert P. H., Billiau A. and Vassalli P. (1989). Monoclonal antibody against interferon *γ* can prevent experimental cerebral malaria and its associated overproduction of tumor necrosis factor. Proceedings of the National Academy of Sciences USA 86, 5572–5574 10.1073/pnas.86.14.5572 PMC297664 2501793

[ref29] Grau G. E., Tacchini-Cottier F., Vesin C., Milon G., Lou J. N., Piguet P. F. and Juillard P. (1993). TNF-induced microvascular pathology: active role for platelets and importance of the LFA-1/ICAM-1 interaction. European Cytokine Network 4, 415–419 7910490

[ref30] Grau G. E., Mackenzie C. D., Carr R. A., Redard M., Pizzolato G., Allasia C., Cataldo C., Taylor T. E. and Molyneux M. E. (2003). Platelet accumulation in brain microvessels in fatal pediatric cerebral malaria. Journal of Infectious Diseases 187, 461–466 12552430 10.1086/367960

[ref31] Hansen D. S. (2012). Inflammatory responses associated with the induction of cerebral malaria: lessons from experimental murine models. PLoS Pathogens 8, e1003045. 10.1371/journal.ppat.1003045 23300435 PMC3531491

[ref32] Hansen D. S., Siomos M. A., Buckingham L., Scalzo A. A. and Schofield L. (2003). Regulation of murine cerebral malaria pathogenesis by CD1d-restricted NKT cells and the natural killer complex. Immunity 18, 391–402 12648456 10.1016/s1074-7613(03)00052-9

[ref33] Hansen D. S., Evans K. J., D'Ombrain M. C., Bernard N. J., Sexton A. C., Buckingham L., Scalzo A. A. and Schofield L. (2005). The natural killer complex regulates severe malarial pathogenesis and influences acquired immune responses to *Plasmodium berghei*-ANKA. Infection and Immunity 73, 2288–2297 15784573 10.1128/IAI.73.4.2288-2297.2005 PMC1087422

[ref34] Hansen D. S., Bernard N. J., Nie C. Q. and Schofield L. (2007). NK cells stimulate recruitment of CXCR3^+^ T cells to the brain during *Plasmodium berghei*-mediated cerebral malaria. Journal of Immunology 178, 5779–5788 10.4049/jimmunol.178.9.5779 17442962

[ref35] Hanum P. S., Hayano M. and Kojima S. (2003). Cytokine and chemokine responses in a cerebral malaria-susceptible or -resistant strain of mice to *Plasmodium berghei*-ANKA infection: early chemokine expression in the brain. International Immunology 15, 633–640 12697663 10.1093/intimm/dxg065

[ref36] Hunt N. H. and Grau G. E. (2003). Cytokines: accelerators and brakes in the pathogenesis of cerebral malaria. Trends in Immunology 24, 491–499 12967673 10.1016/s1471-4906(03)00229-1

[ref37] Jain V., Armah H. B., Tongren J. E., Ned R. M., Wilson N. O., Crawford S., Joel P. K., Singh M. P., Nagpal A. C., Dash A. P., Udhayakumar V., Singh N. and Stiles J. K. (2008). Plasma IP-10, apoptotic and angiogenic factors associated with fatal cerebral malaria in India. Malaria Journal 7, 83. 10.1186/1475-2875-7-83 PMC2405803 18489763

[ref38] John C. C., Opika-Opoka R., Byarugaba J., Idro R. and Boivin M. J. (2006). Low levels of RANTES are associated with mortality in children with cerebral malaria. Journal of Infectious Diseases 194, 837–845. 10.1086/506623 16941352

[ref39] John C. C., Panoskaltsis-Mortari A., Opoka R. O., Park G. S., Orchard P. J., Jurek A. M., Idro R., Byarugaba J. and Boivin M. J. (2008 *a*). Cerebrospinal fluid cytokine levels and cognitive impairment in cerebral malaria. American Journal of Tropical Medicine and Hygiene 78, 198–205 18256412 PMC2254318

[ref40] John C. C., Park G. S., Sam-Agudu N., Opoka R. O. and Bolvin M. J. (2008 *b*). Elevated serum levels of IL-1r*α* in children with *Plasmodium falciparum* malaria are associated with increased severity of disease. Cytokine 41, 204–208. 10.1016/j.cyto.2007.12.008 18282763 PMC2323512

[ref41] Kim C. H., Rott L., Kunkel E. J., Genovese M. C., Andrew D. P., Wu L. J. and Butcher E. C. (2001). Rules of chemokine receptor association with T cell polarization *in vivo*. Journal of Clinical Investigation 108, 1331–1339. 10.1172/jci13543 11696578 PMC209443

[ref42] Kurtzhals J. A. L., Rodrigues O., Addae M., Commey J. O. O., Nkrumah F. K. and Hviid L. (1997). Reversible suppression of bone marrow response to erythropoietin in *Plasmodium falciparum* malaria. British Journal of Haematology 97, 169–174. 10.1046/j.1365-2141.1997.82654.x 9136961

[ref43] Looareesuwan S., Davis T. M. E., Pukrittayakamee S., Supanaranond W., Desakorn V., Silamut K., Krishna S., Boonamrung S. and White N. J. (1991). Erythrocyte survival in severe falciparum malaria. Acta Tropica 48, 263–270. 10.1016/0001-706x(91)90014-b 1674400

[ref44] Looareesuwan S., Suntharasamai P., Webster H. K. and Ho M. (1993). Malaria in splenectomized patients: report of four cases and review. Clinical Infectious Diseases 16, 361–366 8452947 10.1093/clind/16.3.361

[ref45] Lopera-Mesa T. M., Mita-Mendoza N. K., van de Hoef D. L., Doumbia S., Konate D., Doumbouya M., Gu W., Traore K., Diakite S. A. S., Remaley A. T., Anderson J. M., Rodriguez A., Fay M. P., Long C. A., Diakite M. and Fairhurst R. M. (2012). Plasma uric acid levels correlate with inflammation and disease severity in Malian children with *Plasmodium falciparum* malaria. PLoS ONE 7, e46424. 10.1371/journal.pone.0046424 23071567 PMC3465329

[ref46] Lucchi N. W., Peterson D. S. and Moore J. M. (2008). Immunologic activation of human syncytiotrophoblast by *Plasmodium falciparum*. Malaria Journal 7, 42–49. 10.1186/1475-2875-7-42 18312657 PMC2268702

[ref47] Lucchi N. W., Sarr A., Owino S. O., Mwalimu S. M., Peterson D. S. and Moore J. M. (2011). Natural hemozoin stimulates syncytiotrophoblast to secrete chemokines and recruit peripheral blood mononuclear cells. Placenta 32, 579–585. 10.1016/j.placenta.2011.05.003 21632106 PMC3142316

[ref48] Lundie R. J., de Koning-Ward T. F., Davey G. M., Nie C. Q., Hansen D. S., Lau L. S., Mintern J. D., Belz G. T., Schofield L., Carbone F. R., Villadangos J. A., Crabb B. S. and Heath W. R. (2008). Blood-stage *Plasmodium* infection induces CD8^+^ T lymphocytes to parasite-expressed antigens, largely regulated by CD8*α*^+^ dendritic cells. Proceedings of the National Academy of Sciences USA 105, 14509–14514 10.1073/pnas.0806727105 PMC2567226 18799734

[ref49] Luster A. D. (1998). Chemokines – chemotactic cytokines that mediate inflammation. New England Journal of Medicine 338, 436–445 9459648 10.1056/NEJM199802123380706

[ref50] Luster A. D., Greenberg S. M. and Leder P. (1995). The IP-10 chemokine binds to a specific cell-surface heparan-sulfate site shared with platelet factor-4 and inhibits endothelial cell proliferation. Journal of Experimental Medicine 182, 219–231. 10.1084/jem.182.1.219 7790818 PMC2192102

[ref51] Ma N. L., Hunt N. H., Madigan M. C. and ChanLing T. (1996). Correlation between enhanced vascular permeability, up-regulation of cellular adhesion molecules and monocyte adhesion to the endothelium in the retina during the development of fatal murine cerebral malaria. American Journal of Pathology 149, 1745–1762 8909263 PMC1865264

[ref52] Marsh K., Forster D., Waruiru C., Mwangi I., Winstanley M., Marsh V., Newton C., Winstanley P., Warn P., Peshu N., Pasvol G. and Snow R. (1995). Indicators of life-threatening malaria in African children. New England Journal of Medicine 332, 1399–1404. 10.1056/nejm199505253322102 7723795

[ref53] McGregor I. A. (1984). Epidemiology, malaria and pregnancy. American Journal of Tropical Medicine and Hygiene 33, 517–525 6383091 10.4269/ajtmh.1984.33.517

[ref54] Miller L. H., Baruch D. I., Marsh K. and Doumbo O. K. (2002). The pathogenic basis of malaria. Nature 415, 673–679 11832955 10.1038/415673a

[ref55] Miu J., Mitchell A. J., Muller M., Carter S. L., Manders P. M., McQuillan J. A., Saunders B. M., Ball H. J., Lu B., Campbell I. L. and Hunt N. H. (2008). Chemokine gene expression during fatal murine cerebral malaria and protection due to CXCR3 deficiency. Journal of Immunology 180, 1217–1230 10.4049/jimmunol.180.2.1217 18178862

[ref56] Molyneux M. E., Engelmann H., Taylor T. E., Wirima J. J., Aderka D., Wallach D. and Grau G. E. (1993). Circulating plasma receptors for tumour necrosis factor in Malawian children with severe falciparum malaria. Cytokine 5, 604–609 8186373 10.1016/s1043-4666(05)80011-0

[ref57] Mora J. R. and von Andrian U. H. (2006). T-cell homing specificity and plasticity: new concepts and future challenges. Trends in Immunology 27, 235–243. 10.1016/j.it.2006.03.007 16580261

[ref58] Moser B., Wolf M., Walz A. and Loetscher P. (2004). Chemokines: multiple levels of leukocyte migration control. Trends in Immunology 25, 75–84. 10.1016/j.it.2003.12.005 15102366

[ref59] Muehlenbachs A., Fried M., Lachowitzer J., Mutabingwa T. K. and Duffy P. E. (2007). Genome-wide expression analysis of placental malaria reveals features of lymphoid neogenesis during chronic infection. Journal of Immunology 179, 557–565 10.4049/jimmunol.179.1.557 17579077

[ref60] Murphy P. M. (2002). International Union of Pharmacology. Update on chemokine receptor nomenclature. Pharmacological Reviews 54, 227–229. 10.1124/pr.54.2.227 12037138

[ref61] Murray C. J. L., Rosenfeld L. C., Lim S. S., Andrews K. G., Foreman K. J., Haring D., Fullman N., Naghavi M., Lozano R. and Lopez A. D. (2012). Global malaria mortality between 1980 and 2010: a systematic analysis. Lancet 379, 413–431 22305225 10.1016/S0140-6736(12)60034-8

[ref62] Neres R., Marinho C. R. F., Goncalves L. A., Catarino M. B. and Penha-Goncalves C. (2008). Pregnancy outcome and placenta pathology in *Plasmodium berghei*-ANKA-infected mice reproduce the pathogenesis of severe malaria in pregnant women. PLoS ONE 3, e1608. 10.1371/journal.pone.0001608 18270595 PMC2229663

[ref63] Nie C. Q., Bernard N. J., Norman M. U., Amante F. H., Lundie R. J., Crabb B. S., Heath W. R., Engwerda C. R., Hickey M. J., Schofield L. and Hansen D. S. (2009). IP-10-mediated T cell homing promotes cerebral inflammation over splenic immunity to malaria infection. PLoS Pathogens 5, e1000369 19343215 10.1371/journal.ppat.1000369 PMC2658824

[ref64] Nitcheu J., Bonduelle O., Combadiere C., Tefit M., Seilhean D., Mazier D. and Combadiere B. (2003). Perforin-dependent brain-infiltrating cytotoxic CD8^+^ T lymphocytes mediate experimental cerebral malaria pathogenesis. Journal of Immunology 170, 2221–2228 10.4049/jimmunol.170.4.2221 12574396

[ref65] Ochiel D. O., Awandare G. A., Keller C. C., Hittner J. B., Kremsner P. G., Weinberg J. B. and Perkins D. J. (2005). Differential regulation of beta-chemokines in children with *Plasmodium falciparum* malaria. Infection and Immunity 73, 4190–4197. 73/7/4190 10.1128/IAI.73.7.4190-4197.2005 15972509 PMC1168587

[ref66] Ordi J., Ismail M. R., Ventura P. J., Kahigwa E., Hirt R., Cardesa A., Alonso P. L. and Menendez C. (1998). Massive chronic intervillositis of the placenta associated with malaria infection. American Journal of Surgical Pathology 22, 1006–1011. 10.1097/00000478-199808000-00011 9706981

[ref67] Ordi J., Menendez C., Ismail M. R., Ventura P. J., Palacin A., Kahigwa E., Ferrer B., Cardesa A. and Alonso P. L. (2001). Placental malaria is associated with cell-mediated inflammatory responses with selective absence of natural killer cells. Journal of Infectious Diseases 183, 1100–1107. 10.1086/319295 11237836

[ref68] Patnaik J. K., Das B. S., Mishra S. K., Mohanty S., Satpathy S. K. and Mohanty D. (1994). Vascular clogging, mononuclear cell margination, and enhanced vascular-permeability in the pathogenesiss of human cerebral malaria. American Journal of Tropical Medicine and Hygiene 51, 642–647 7985757

[ref69] Pongponratn E., Turner G. D., Day N. P., Phu N. H., Simpson J. A., Stepniewska K., Mai N. T., Viriyavejakul P., Looareesuwan S., Hien T. T., Ferguson D. J. and White N. J. (2003). An ultrastructural study of the brain in fatal *Plasmodium falciparum* malaria. American Journal of Tropical Medicine and Hygiene 69, 345–359 14640492

[ref70] Porta J., Carota A., Pizzolato G. P., Wildi E., Widmer M. C., Margairaz C. and Grau G. E. (1993). Immunopathological changes in human cerebral malaria. Clinical Neuropathology 12, 142–146 8100753

[ref71] Renia L., Potter S. M., Mauduit M., Rosa D. S., Kayibanda M., Deschemin J. C., Snounou G. and Gruner A. C. (2006). Pathogenic T cells in cerebral malaria. International Journal for Parasitology 36, 547–554 16600241 10.1016/j.ijpara.2006.02.007

[ref72] Rodrigues-Duarte L., de Moraes L. V., Barboza R., Marinho C. R. F., Franke-Fayard B., Janse C. J. and Penha-Goncalves C. (2012). Distinct placental malaria pathology caused by different *Plasmodium berghei* lines that fail to induce cerebral malaria in the C57BL/6 mouse. Malaria Journal 11, 231. 10.1186/1475-2875-11-231 PMC3485172 22799533

[ref73] Rogerson S. J., Brown H. C., Pollina E., Abrams E. T., Tadesse E., Lema V. M. and Molyneux M. E. (2003 *a*). Placental tumor necrosis factor *α* but not *γ* interferon is associated with placental malaria and low birth weight in Malawian women. Infection and Immunity 71, 267–270 12496175 10.1128/IAI.71.1.267-270.2003 PMC143363

[ref74] Rogerson S. J., Pollina E., Getachew A., Tadesse E., Lema V. M. and Molyneux M. E. (2003 *b*). Placental monocyte infiltrates in response to *Plasmodium falciparum* malaria infection and their association with adverse pregnancy outcomes. American Journal of Tropical Medicine and Hygiene 68, 115–119 12556159

[ref75] Rollins B. J. (1997). Chemokines. Blood 90, 909–928 9242519

[ref76] Romagnani P., Annunziato F., Lasagni L., Lazzeri E., Beltrame C., Francalanci M., Uguccioni M., Galli G., Cosmi L., Maurenzig L., Baggiolini M., Maggi E., Romagnani S. and Serio M. (2001). Cell cycle-dependent expression of CXC chemokine receptor 3 by endothelial cells mediates angiostatic activity. Journal of Clinical Investigations 107, 53–63. 10.1172/jci9775 PMC198541 11134180

[ref77] Rot A. (1992). Endothelial cell binding of NAP-1/IL-8 – role in neutrophil emigration. Immunology Today 13, 291–294. 10.1016/0167-5699(92)90039-a 1510812

[ref78] Rot A. and von Andrian U. H. (2004). Chemokines in innate and adaptive host defense: basic chemokinese grammar for immune cells. Annual Review of Immunology 22, 891–928. 10.1146/annurev.immunol.22.012703.104543 15032599

[ref79] Ryg-Cornejo V., Nie C. Q., Bernard N. J., Lundie R. J., Evans K. J., Crabb B. S., Schofield L. and Hansen D. S. (2013). NK cells and conventional dendritic cells engage in reciprocal activation for the induction of inflammatory responses during *Plasmodium berghei*-ANKA infection. Immunobiology 218, 263–271. 10.1016/j.imbio.2012.05.018 22704523

[ref80] Sallusto F., Lanzavecchia A. and Mackay C. R. (1998). Chemokines and chemokine receptors in T-cell priming and Th1/Th2-mediated responses. Immunology Today 19, 568–574 9864948 10.1016/s0167-5699(98)01346-2

[ref81] Sallusto F., Mackay C. R. and Lanzavecchia A. (2000). The role of chemokine receptors in primary, effector, and memory immune responses. Annual Review of Immunology 18, 593–620. 10.1146/annurev.immunol.18.1.593 10837070

[ref82] Sarfo B. Y., Singh S., Lillard J. W., Quarshie A., Gyasi R. K., Armah H., Adjei A. A., Jolly P. and Stiles J. K. (2004). The cerebral-malaria-associated expression of RANTES, CCR3 and CCR5 in post-mortem tissue samples. Annals of Tropical Medicine and Parasitology 98, 297–303 15119976 10.1179/000349804225003271

[ref83] Sarfo B. Y., Armah H. B., Irune I., Adjei A. A., Olver C. S., Singh S., Lillard J. W. Jr. and Stiles J. K. (2005). *Plasmodium yoelii* 17XL infection up-regulates RANTES, CCR1, CCR3 and CCR5 expression, and induces ultrastructural changes in the cerebellum. Malaria Journal 4, 63. 10.1186/1475-2875-4-63 16359553 PMC1343570

[ref84] Sayles P. C., Yanez D. M. and Wassom D. L. (1993). *Plasmodium yoelii*: splenectomy alters the antibody responses of infected mice. Experimental Parasitology 76, 377–384 8513875 10.1006/expr.1993.1046

[ref85] Schofield L. and Grau G. E. (2005). Immunological processes in malaria pathogenesis. Nature Reviews Immunology 5, 722–735 10.1038/nri1686 16138104

[ref86] Serbina N. V., Jia T., Hohl T. M. and Pamer E. G. (2008). Monocyte-mediated defense against microbial pathogens. Annual Reviews Immunology 26, 421–452. 10.1146/annurev.immunol.26.021607.090326 PMC2921669 18303997

[ref87] Sexton A. C., Good R. T., Hansen D. S., D'Ombrain M. C., Buckingham L., Simpson K. and Schofield L. (2004). Transcriptional profiling reveals suppressed erythropoiesis, up-regulated glycolysis, and interferon-associated responses in murine malaria. Journal of Infectious Diseases 189, 1245–1256 15031794 10.1086/382596

[ref88] Sponaas A. M., Freitas do Rosario A. P., Voisine C., Mastelic B., Thompson J., Koernig S., Jarra W., Renia L., Mauduit M., Potocnik A. J. and Langhorne J. (2009). Migrating monocytes recruited to the spleen play an important role in control of blood stage malaria. Blood 114, 5522–5531. 10.1182/blood-2009-04-217489 19837977

[ref89] Srivastava K., Cockburn I. A., Swaim A., Thompson L. E., Tripathi A., Fletcher C. A., Shirk E. M., Sun H., Kowalska M. A., Fox-Talbot K., Sullivan D., Zavala F. and Morrell C. N. (2008). Platelet factor 4 mediates inflammation in experimental cerebral malaria. Cell Host Microbe 4, 179–187. 10.1016/j.chom.2008.07.003 18692777 PMC2603442

[ref90] Srivastava K., Field D. J., Aggrey A., Yamakuchi M. and Morrell C. N. (2010). Platelet factor 4 regulation of monocyte KLF4 in experimental cerebral malaria. PLoS ONE 5, e10413. 10.1371/journal.pone.0010413 20454664 PMC2862712

[ref91] Steketee R. W., Nahlen B. L., Parise M. E. and Menendez C. (2001). The burden of malaria in pregnancy in malaria-endemic areas. American Journal of Tropical Medicine and Hygiene 64, 28–35 11425175 10.4269/ajtmh.2001.64.28

[ref92] Suguitan A. L., Leke R. G. F., Fouda G., Zhou A. N., Thuita L., Metenou S., Fogako J., Megnekou R. and Taylor D. W. (2003). Changes in the levels of chemokines and cytokines in the placentas of women with *Plasmodium falciparum* malaria. Journal of Infectious Diseases 188, 1074–1082 14513430 10.1086/378500

[ref93] Tanaka Y., Adams D. H. and Shaw S. (1993). Proteoglycans on endothelial cells present adhesion-inducing cytokines to leukocytes. Immunology Today 14, 111–115. 10.1016/0167-5699(93)90209-4 8466625

[ref94] Taylor T. E., Fu W. J., Carr R. A., Whitten R. O., Mueller J. S., Fosiko N. G., Lewallen S., Liomba N. G. and Molyneux M. E. (2004). Differentiating the pathologies of cerebral malaria by postmortem parasite counts. Nature Medicine 10, 143–145 10.1038/nm986 14745442

[ref95] Tripathi A. K., Sha W., Shulaev V., Stins M. F. and Sullivan D. J. Jr. (2009). *Plasmodium falciparum*-infected erythrocytes induce NF-*κ*B regulated inflammatory pathways in human cerebral endothelium. Blood 114, 4243–4252. 10.1182/blood-2009-06-226415 19713460 PMC2925626

[ref96] Van den Steen P. E., Deroost K., Aelst I. V., Geurts N., Martens E., Struyf S., Nie C. Q., Hansen D. S., Matthys P., Damme J. V. and Opdenakker G. (2008). CXCR3 determines strain susceptibility to murine cerebral malaria by mediating T lymphocyte migration toward IFN-*γ*-induced chemokines. European Journal of Immunology 38, 1082–1095 18383042 10.1002/eji.200737906

[ref97] van Hensbroek M. B., Palmer A., Onyiorah E., Schneider G., Jaffar S., Dolan G., Memming H., Frenkel J., Enwere G., Bennett S., Kwiatkowski D. and Greenwood B. (1996). The effect of a monoclonal antibody to tumor necrosis factor on survival from childhood cerebral malaria. Journal of Infectious Diseases 174, 1091–1097 8896514 10.1093/infdis/174.5.1091

[ref98] Viebig N. K., Wulbrand U., Forster R., Andrews K. T., Lanzer M. and Knolle P. A. (2005). Direct activation of human endothelial cells by *Plasmodium falciparum*-infected erythrocytes. Infection and Immunity 73, 3271–3277. 10.1128/iai.73.6.3271-3277.2005 15908351 PMC1111820

[ref99] Villegas-Mendez A., Greig R., Shaw T. N., de Souza J. B., Gwyer Findlay E., Stumhofer J. S., Hafalla J. C., Blount D. G., Hunter C. A., Riley E. M. and Couper K. N. (2012). IFN-*γ*-producing CD4^+^ T cells promote experimental cerebral malaria by modulating CD8^+^ T cell accumulation within the brain. Journal of Immunology 189, 968–979. 10.4049/jimmunol.1200688 PMC3393641 22723523

[ref100] Weidanz W. P., LaFleur G., Brown A., Burns J. M. Jr., Gramaglia I. and van der Heyde H. C. (2010). *γδ* T cells but not NK cells are essential for cell-mediated immunity against *Plasmodium chabaudi* malaria. Infection and Immunity 78, 4331–4340. 10.1128/iai.00539-10 20660608 PMC2950347

[ref101] Weninger W., Crowley M. A., Manjunath N. and von Andrian U. H. (2001). Migratory properties of naive, effector, and memory CD8^+^ T cells. Journal of Experimental Medicine 194, 953–966. 10.1084/jem.194.7.953 11581317 PMC2193483

[ref102] Were T., Ouma C., Otieno R. O., Orago A. S. S., Ong'echa J. M., Vulule J. M., Keller C. C. and Perkins D. J. (2006). Suppression of RANTES in children with *Plasmodium falciparum* malaria. Haematologica – the Hematology Journal 91, 1396–1399 PMC13402947 17018392

[ref103] Were T., Davenport G. C., Yamo E. O., Hittner J. B., Awandare G. A., Otieno M. F., Ouma C., Orago A. S. S., Vulule J. M., Ong'echa J. M. and Perkins D. J. (2009). Naturally acquired hemozoin by monocytes promotes suppression of RANTES in children with malarial anemia through an IL-10-dependent mechanism. Microbes and Infection 11, 811–819. 10.1016/j.micinf.2009.04.021 19427395 PMC2745194

[ref104] White N. J. and Ho M. (1992). The pathophysiology of malaria. Advances in Parasitology 31, 83–173 1496930 10.1016/s0065-308x(08)60021-4

[ref105] Wilson N. O., Jain V., Roberts C. E., Lucchi N., Joel P. K., Singh M. P., Nagpal A. C., Dash A. P., Udhayakumar V., Singh N. and Stiles J. K. (2011). CXCL4 and CXCL10 predict risk of fatal cerebral malaria. Disease Markers 30, 39–49. 10.3233/dma-2011-0763 21508508 PMC3260027

[ref106] Wilson N. O., Solomon W., Anderson L., Patrickson J., Pitts S., Bond V., Liu M. and Stiles J. K. (2013). Pharmacologic inhibition of CXCL10 in combination with anti-malarial therapy eliminates mortality associated with murine model of cerebral malaria. PLoS ONE 8, e60898. 10.1371/journal.pone.0060898 23630573 PMC3618178

[ref107] Xie H. J., Lim Y. C., Luscinskas F. W. and Lichtman A. H. (1999). Acquisition of selectin binding and peripheral homing properties by CD4^+^ and CD8^+^ T cells. Journal of Experimental Medicine 189, 1765–1775. 10.1084/jem.189.11.1765 10359580 PMC2193075

[ref108] Yanez D. M., Manning D. D., Cooley A. J., Weidanz W. P. and van der Heyde H. C. (1996). Participation of lymphocyte subpopulations in the pathogenesis of experimental murine cerebral malaria. Journal of Immunology 157, 1620–1624 8759747

[ref109] Yap G. S. and Stevenson M. M. (1994). Differential requirements for an intact spleen in induction and expression of B-cell-dependent immunity to *Plasmodium chabaudi* AS. Infection and Immunity 62, 4219–4225 7927677 10.1128/iai.62.10.4219-4225.1994 PMC303098

[ref110] Yoshie O., Imai T. and Nomiyama H. (1997). Novel lymphocyte-specific CC chemokines and their receptors. Journal of Leukocyte Biology 62, 634–644 9365118 10.1002/jlb.62.5.634

